# Fecal Microbiota Transplantation Alters the Outcome of Hepatitis B Virus Infection in Mice

**DOI:** 10.3389/fcimb.2022.844132

**Published:** 2022-05-04

**Authors:** Junzhong Wang, Xin Zhou, Xiaoran Li, Weina Guo, Qingfeng Zhu, Bin Zhu, Yinping Lu, Xin Zheng, Dongliang Yang, Baoju Wang

**Affiliations:** Department of Infectious Diseases, Union Hospital, Tongji Medical College, Huazhong University of Science and Technology, Wuhan, China

**Keywords:** gut microbiota, fecal microbiota transplantation, bacteria colonization, HBV, T cells response

## Abstract

The susceptibility of mice to hepatitis B virus (HBV) infection depends on their genetic background. The gut microbiota modulates the antiviral immune response in the liver and plays a protective role against HBV infection. However, whether HBV infection outcomes depend on the gut microbiota remains unclear. In this study, we assessed the gut microbiota composition in naïve BALB/c and C57BL/6 mice using 16S rRNA gene sequencing. The gut microbiota in BALB/c mice was depleted using broad-spectrum antibiotics (ABX) and then reconstituted with fecal microbiota from naïve BALB/c or C57BL/6 mice to evaluate the effect of fecal microbiota transplantation (FMT) on the outcomes of and immune response to HBV infection. We found that HBV infection outcomes and the gut microbiota composition differed between BALB/c and C57BL/6 mice. Commensal bacteria from the fecal microbiota selectively colonized the guts of ABX-treated BALB/c mice. Mice receiving fecal microbiota from BALB/c or C57BL/6 mice displayed different HBV infection outcomes. The fecal microbiota from C57BL/6 mice induced immune tolerance in the liver and prolonged HBV infection. In conclusion, HBV infection outcomes in mice are determined by the host genetic background and gut microbiota composition. Reconstitution of the gut microbiota by FMT can alter the susceptibility to HBV infection in mice.

## Introduction

The human intestinal tract is colonized by trillions of commensal bacteria that can modulate host immunity. Increasing evidence indicates that the gut microbiota influences the susceptibility of an organism to infectious diseases. The commensal microbiota acts as a barrier that prevents the invasion of pathogens in the gastrointestinal tract and thus plays a protective role. Moreover, certain components of the gut microbiota can regulate the development of immune progenitors in the bone marrow, which in turn affects the functionality of resident immune cells and modulates the systemic immune response to infection. The colonization of gut microbiota contributes to the development and maturation of immune system in gut and extra-intestinal organs. The germ-free mice are characterized with lower T cell numbers. Colonization of mouse-segmented filamentous bacteria partially restores T cell numbers, which provide better protection against *Salmonella* infection. Interestingly, evidences from mono-colonized mice showed that diverse microbial species can induce similar immune responses, while closely related bacterial species often differ in terms of immune activation. The immunological niche in gut is composed by innate and adaptive immune systems. Both the specific species colonization and gut dysbiosis can modify the balance of immunological niche and microbiota, which may promote the infection and inflammation (Pamer, 2017; Cianci et al., 2018).

HBV is a hepadnavirus that infects hepatocytes in humans and chimpanzees. HBV infection outcomes are influenced by the age of the host. In immunocompetent adults, HBV infection spontaneously resolves in more than 95% of cases. However, in infants and young children, 95% of infection cases develop into persistent infections. Clinical presentations of patients with chronic HBV infection are variable, ranging from asymptomatic or mild hepatitis to liver cirrhosis, liver failure, and hepatocellular carcinoma (Ganem and Prince, 2004). It remains unclear whether the susceptibility to HBV infection is affected by the gut microbiota composition. However, in patients with HBV infection, gut microbiota dysbiosis is observed and is associated with the disease stage. In patients with HBV-related liver disease, the abundance and diversity of the gut microbiota are significantly decreased, and the metabolic profile is shifted (Qin et al., 2014; Wang et al., 2017; Zeng et al., 2020). Combined with long-term antiviral treatment, fecal microbiota transplantation **(**FMT) can alter the gut microbiota composition and induce hepatitis e antigen (HBeAg) decline or clearance in patients with chronic hepatitis B (Ren et al., 2017).

In mice, the age-related immune response to HBV infection also depends on the establishment of the gut microbiota. In young C3H/HeN mice, the gut microbiota has not yet reached equilibrium and cannot trigger an adaptive immune response to control HBV infection (Chou et al., 2015). HBV infection can delay commensal bacterial colonization and development in mice, and the dynamic developmental progression of the gut microbiota differs between transiently and persistently infected mice (Zhu et al., 2019). After depletion of the gut microbiota with broad-spectrum antibiotics (ABX), commensal bacteria and their components can translocate to the liver and impair the T-cell response against HBV, leading to prolonged HBV infection in mice (Guo et al., 2021).

The genetic background also is a critical factor in determining HBV infection outcomes in mice. Following the hydrodynamic injection of an HBV plasmid, pAAV/HBV1.2, HBV infection persisted for more than six months in adult C57BL/6 mice but was resolved within two weeks in adult BALB/c mice (Huang et al., 2006). A further study confirmed that HBV infection persisted only in C57BL/6J mice, but not in the C57BL/6N mice (Wang et al., 2018). The gut microbiota composition differs among mouse strains, and the differences are related to both genetic and environmental factors (Hufeldt et al., 2010). And cohousing can induce horizontal transmission of gut microbiota between different strains of mice (Caruso et al., 2019). Whether differences in the outcomes of HBV infection are determined by differences in the gut microbiota between BALB/c and C57BL/6 mice is not well known. In this study, we depleted the gut microbiota in BALB/c mice using antibiotics and then reconstituted it by FMT using fecal microbiota from BALB/c or C57BL/6 mice. We aimed to investigate whether the donor’s fecal microbiota can modulate the antiviral immune response and determine the outcome of HBV infection in recipient BALB/c mice.

## Material and Methods

### Animals

Six to eight-week-old male BALB/c and C57BL/6 (C57BL/6J substrain was used in this study) mice were purchased from Hunan SJA Laboratory Animal Co., Ltd. (Hunan, China) and were housed in a specific pathogen-free facility at Huazhong University of Science and Technology. In the new facility, a 12-hour light/12-hour dark cycle was used, temperature of 20-26°C and 40%-60% humidity were kept. The mice food was composed of 12% fats, 20.6 proteins, and 67.4% carbohydrates.

This study was carried out according to the Guide for the Care and Use of Laboratory Animals of the National Institutes of Health and was approved by the Institutional Animal Care and Use Committee at Tongji Medical College, Huazhong University of Science and Technology (permit number: S814).

### Gut Microbiota Depletion and FMT

To establish substantial and sustained microbiota engraftment from donor mice into recipient mice, BALB/c mice were subjected to a streamlined ABX conditioning regimen, as described previously (Staley et al., 2017). Two antibiotic cocktails were used; cocktail 1 consisted of three highly absorptive antibiotics: ampicillin, cefoperazone sodium salt, and clindamycin hydrochloride, and cocktail 2 comprised three lower-degree absorptive antibiotics: ertapenem (Invanz), neomycin sulfate, and vancomycin hydrochloride. The antibiotics were dissolved in drinking water at a concentration of 1 mg/mL. BALB/c mice were treated with antibiotic cocktail 1 on days 0 to 7, with cocktail 2 on days 9 to 18, and then switched back to cocktail 1 during days 18 to 25 (**Figure 3A**).

To reconstitute the gut microbiota after ABX treatment, FTM was carried out as described previously (Lei et al., 2016; Villarino et al., 2016; Rosshart et al., 2017). Fresh fecal pellets from naïve C57BL/6 or BALB/c mice were collected and resuspended in 1 mL of sterile PBS. After filtration through a 70-μm cell strainer, the fecal material was transplanted into recipient BALB/c mice using daily oral gavages on days 27 to 34 (**Figure 3A**).

### HBV Infection Mouse Model

A previously described (Huang et al., 2006; Zhu et al., 2019; Guo et al., 2021), well-established HBV infection mouse model was used in this study. Briefly, 10 μg of HBV plasmid, pAAV/HBV1.2, was diluted in PBS and injected at 0.1 mL/g of mouse body weight *via* the tail vein within 5–8 s. At the indicated time points, blood samples were collected from the mice, and sera were diluted 1:10 in PBS. HBsAg, HBsAb, HBeAg, HBeAb, and HBcAb levels were measured using an ELISA kit (Kehua, Shanghai, China). Serum HBV DNA titers were assessed using a qPCR kit (Sigma-Aldrich, St. Louis, MO, USA).

### Immunohistochemical Analysis

Liver samples were fixed in 4% paraformaldehyde and embedded in paraffin, as described previously (Wang et al., 2014). A rabbit anti-HBV core antigen (HBcAg) polyclonal antibody (Dako, Copenhagen, Denmark) was used for IHC staining, and the sections were visualized using a DAKO EnVision™ Detection System (Dako).

### 16S rRNA Gene Sequencing

Fecal pellets were harvested and stored at –80°C. Bacterial DNA was extracted using an E.Z.N.A. Soil DNA Kit (Omega Bio-tek, Norcross, GA, USA). 16S rRNA gene sequencing was performed as described previously (Zhu et al., 2019; Guo et al., 2021), using primers to amplify the V3-V4 hypervariable regions of the bacterial 16S rRNA gene (338F, 5′-ACTCCTACGGGAGGCAGCAG-3′ and 806R 5′-GGACTACHVGGGTWTCTAAT-3′). Amplicons were purified and paired-end sequenced (2 × 300) using an Illumina MiSeq System (Illumina, San Diego, CA, USA). The sequencing data have been deposited into the NCBI Sequence Read Archive database (accession No.: SRP174629).

### Flow Cytometry

Mouse livers were perfused with 10 mL of PBS and homogenized in Hanks’ buffer. The homogenate was resuspended in 36.5% Percoll (Sigma-Aldrich), and lymphocytes were isolated by density gradient centrifugation. The lymphocytes were cultured in RPMI 1640 medium and stimulated with CD8+ T-cell epitope (L^d^-HBV S28–39 epitope, IPQSLDSWWTSL, 10 μg/mL) as described previously (Schirmbeck et al., 2001; Sette et al., 2001). The following antibodies were used for cell-surface staining and intracellular cytokine staining: BV421-anti-CD8, APC-Cy7-anti-CD4, PE-anti-CTLA4, and PE-Cy7-anti-PD1 (eBioscience, San Diego, CA, USA). For intracellular cytokine staining, APC-anti-IFN-γ, PerCP-Cy5.5-anti-IL-10, and FITC-anti-TNF-α (BioLegend, San Diego, USA) were used. Dead cells were excluded by staining with Fixable Viability Dye eFluor 506 (eBioscience). Samples were analyzed using a FACSCanto II flow cytometer (BD Biosciences, San Jose, CA, USA). Data were analyzed using FlowJo (version 10.0; TreeStar, Ashland, OR, USA).

### Statistical Analysis

Data are presented as the mean ± SD. For the comparison analyzing between two groups, two-tailed unpaired Student *t*-test was used for data with normal distribution, Mann-Whitney test were used for data with non-normal distribution. Means of multiple groups were compared using one-way ANOVA followed by Tukey’s multiple comparison tests. Kaplan–Meier survival analysis was used to compare the frequency of HBsAg-positive mice. *P* < 0.05 was considered significant. Statistical analyses were performed using SPSS (version 12.0; SPSS Inc., Chicago, IL, USA).

## Results

### HBV Infection Outcomes Depend on the Mouse Genetics Background

**Figure 1:**
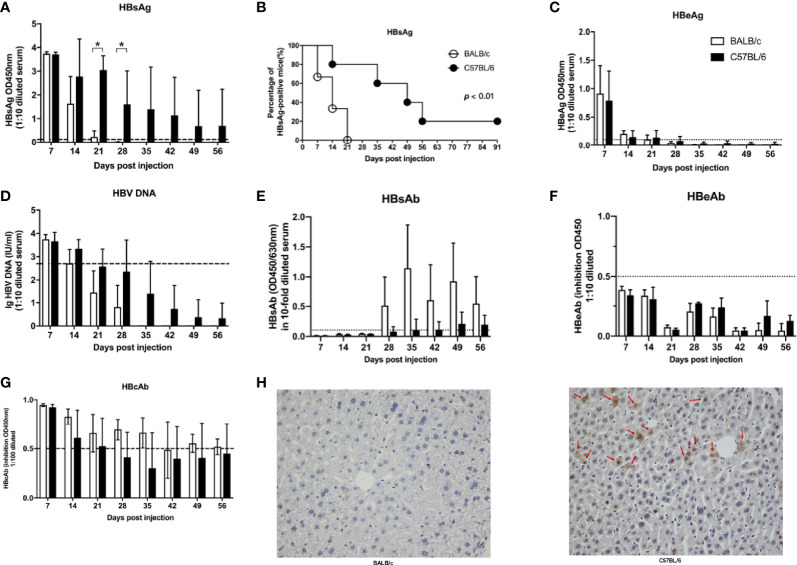
Host gene background determined the outcomes of HBV infection in mice. BALB/c and C57B/6 mice were hydrodynamically injected with pAAV/HBV1.2. The levels of HBsAg **(A)**, HBeAg **(C)**, HBV DNA **(D)**, HBsAb **(E)**, HBeAb **(F)**, HBcAb **(G)** in sera were measured at the indicated times. The cutoff values are indicated by dotted lines. **(B)** Kaplan–Meier curve showing the percentage of HBsAg-positive mice. **(H)** Mice were euthanized at 56 dpi, and the expression of HBcAg in the liver was measured by IHC. The HBcAg positive hepatocytes was labeled with red arrows. n=10/group, *P < 0.05.

To confirm that the genetic background of the host influences the HBV infection outcome, BALB/c and C57BL/6 mouse HBV infection models were established. Serum levels of HBV surface antigen (HBsAg) in BALB/c mice were lower than those in C57BL/6 mice at 21 days post infection (dpi) and 28 dpi (**Figure 1A**). HBsAg was cleared from the serum within 21 days in BALB/c mice but persisted for more than 91 days in 20% of the C57BL/6 mice (**Figure 1B**). HBeAg and HBV DNA were detected in sera at 7 dpi but were undetectable after 21 dpi in both groups (**Figures 1C, D**). HBV surface antibody (HBsAb) was detectable in sera after 28 dpi, and HBsAb levels were slightly, albeit not significantly, higher in BALB/c mice than in C57BL/6 mice (**Figure 1E**). HBV e antibody (HBeAb) was undetectable in sera of both BALB/c and C57BL/6 mice (**Figure 1F**). HBV core antibody (HBcAb) was detected at 7 dpi and gradually decreased in both BALB/c and C57BL/6 mice, with no significant differences (**Figure 1G**). At 56 dpi, HBV core antigen (HBcAg) was detected in the livers of C57BL/6 mice but not in those of BALB/c mice (**Figure 1H**). These results indicated that HBV infection outcomes differ depending on the host genetic background. HBV infection tends to be transient in BALB/c mice but persistent in C57BL/6 mice.

### The Gut Microbiota Composition Differs Between BALB/c and C57BL/6 Mice

**Figure 2:**
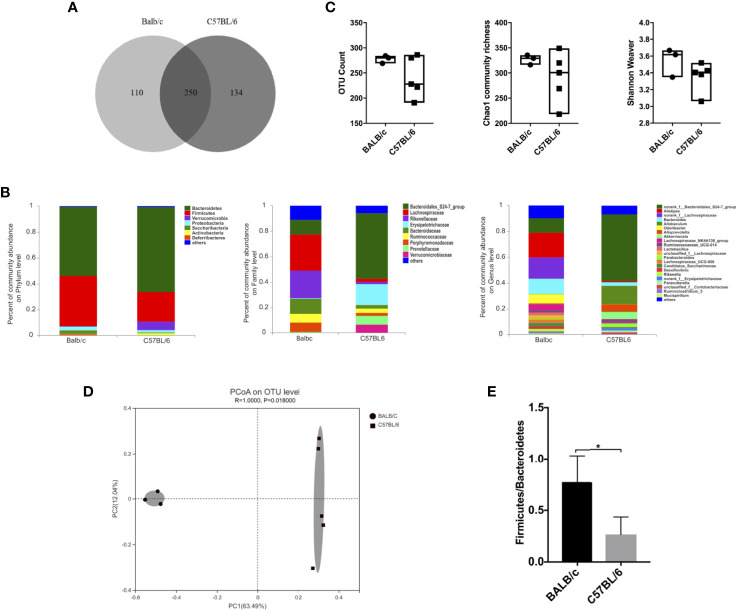
Comparison of the gut microbiota between naïve BALB/c and C57BL/6 mice. 16S rRNA in fecal pellets from naïve BALB/c and C57BL/6 mice was sequenced. **(A)** Venn diagram showing shared and unique OTUs. **(B)** Gut microbiota composition at the phylum, family, and genus levels. **(C)** α-Diversity based on OTU counts, Chao1 community richness, and the Shannon–Weaver index. **(D)** β-Diversity based on Bray-Curtis dissimilarity. **(E)** Firmicutes/Bacteroidetes ratio calculated on the basis of the abundance of each bacterial species. n = 3 in BALB/c group, n = 5 in C57BL/6 group, *P < 0.05.

Given that the gut microbiota affects HBV infection outcomes in mice (Chou et al., 2015), we comparatively investigated the gut microbiota composition in naïve adult BALB/c and C57BL/6 mice. Fecal pellets were collected and analyzed by 16S rRNA sequencing. We identified 494 operational taxonomic units (OTUs); 250 OTUs were shared by both BALB/c mice and C57BL/6 mice, 110 OTUs were unique to BALB/c mice, and 134 OTUs were unique to C57BL/6 mice (**Figure 2A**).

The gut microbiota composition in BALB/c and C57BL/6 mice was assessed at the phylum, family, and genus levels (**Figure 2B**). At the phylum level, the abundance of Firmicutes was higher, whereas those of Bacteroidetes and Verrucomicrobia were lower in BALB/c mice than in C57BL/6 mice. At the family level, the abundances of Lachnospiraceae, Rikenellaceae, Bacteroidaceae, Ruminococcaceae, and Porphyromonadaceae were higher, whereas those of Bacteroidales_S24-7_group, Erysipelotrichaceae, and Prevotellaceae were lower in BALB/c mice than in C57BL/6 mice. At the genus level, the abundances of *Alistipes*, *norank_f_Lachnospiraceae*, *Bacteroides*, and *Odoribacter* were higher, whereas those of *norank_f_Bacteroidales_S24-7_group*, *Candidatus Saccharimonas*, *Rikenella*, and *Parabacteroides* were lower in BALB/c mice than in C57BL/6 mice.

As for diversity, no significant differences were observed in OTU counts, Chao1 community richness, and Shannon–Weaver index values, indicating that the α-diversity was comparable between BALB/c mice and C57BL/6 mice (**Figure 2C**). However, the β-diversity differed significantly between BALB/c mice and C57BL/6 mice as determined by Bray-Curtis dissimilarity (**Figure 2D**). The Firmicutes/Bacteroidetes ratio was significantly higher in BALB/c mice than in C57BL/6 mice (**Figure 2E**). The ratio of *Faecalibacterium prausnitzii*/*Escherichia coli* at species level was comparable (data not shown). These results demonstrated that the gut microbiota composition is influenced by the genetic background in mice.

### Gut Microbiota Reconstitution by FMT After ABX Treatment in BALB/c Mice

**Figure 3:**
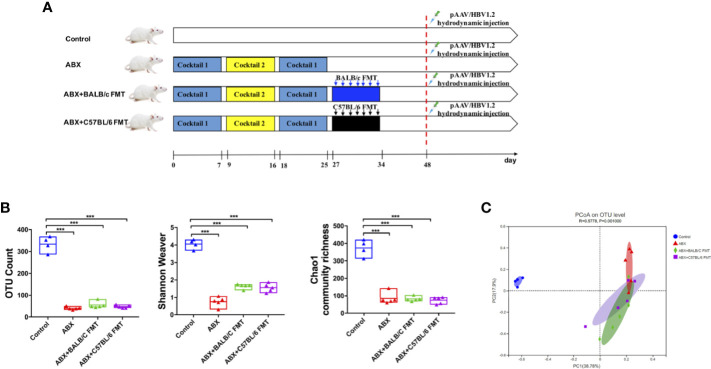
Gut microbiota depletion by ABX treatment in BALB/c mice. **(A)** Schedule of the experiment. BALB/c mice were sequentially treated with two ABX cocktails. After treatment withdrawal, fecal microbiota from naïve BALB/c or C57BL/6 mice was transplanted into recipient mice by seven consecutive daily oral gavages. After reconstitution for 2 weeks, the mice were hydrodynamically injected with pAAV/HBV1.2. On day 25, the gut microbiota diversity was assessed based on OTU counts, Chao1 community richness, and the Shannon–Weaver index **(B)** and Bray-Curtis dissimilarity **(C)**. n = 4 in control group. n = 5 in ABX, ABX+BALB/c FMT, ABX+C57BL/6 groups. ***P < 0.001.

**Figure 4:**
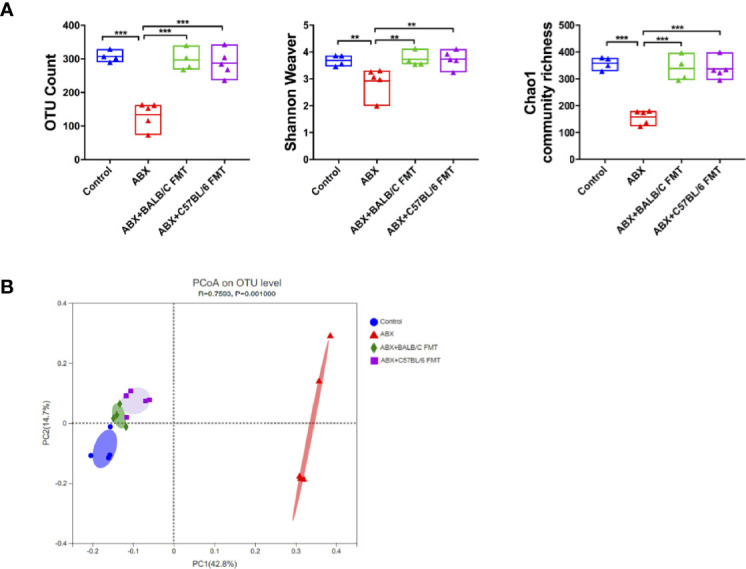
FMT reconstitutes the gut microbiota in BALB/c mice. After FMT (on day 35 as indicated in **Figure 3A**), the gut microbiota diversity was assessed based on OTU counts, Chao1 community richness, and the Shannon–Weaver index **(A)** and Bray-Curtis dissimilarity **(B)**. n = 4 in control group. n = 5 in ABX, ABX+BALB/c FMT, ABX+C57BL/6 groups. **P < 0.01, ***P < 0.001.

To confirm that HBV infection outcomes in BALB/c and C57BL/6 mice are influenced by the gut microbiota composition, we depleted the gut microbiota in BALB/c mice using ABX and then reconstituted it with fecal microbiota from naïve adult BALB/c or C57BL/6 mice (**Figure 3A**). After ABX treatment, fecal pellets were collected and analyzed by 16S rRNA sequencing on day 25. The OTU counts, Chao1 community richness, and Shannon–Weaver index value were significantly lower in ABX-treated mice than in control mice (**Figure 3B**), and Bray-Curtis dissimilarity indicated that the microbiota composition was significantly shifted after ABX treatment (**Figure 3C**; ANOSIM, *r* = 0.5778, *P* = 0.001; PERMANOVA, *pseudo-F* = 5.87, *P* = 0.001). On days 27 to 34, fecal microbiota from naïve adult BALB/c or C57BL/6 mice was transplanted into recipient mice (**Figure 3A**). On day 35, OTU counts, Chao1 community richness, and Shannon–Weaver index values in mice of the ABX+BALB/c FMT and ABX+C57BL/6 FMT groups were restored to the levels in the control mice and were significantly higher than those in mice of the ABX group (**Figure 4A**). The Bray-Curtis dissimilarity was also restored after FMT in both the ABX+BALB/c FMT and ABX+C57BL/6 FMT groups (**Figure 4B**). These results indicated that ABX-depleted gut microbiota in BALB/c mice can be reconstituted by transplantation of commensal bacteria from BALB/c or C57BL/6 mice.

### Gut Microbiome Profiles Shift After FTM

**Figure 5:**
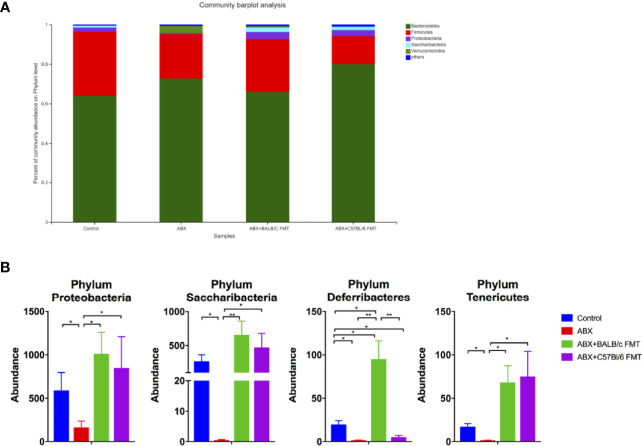
Comparison of the gut microbiota composition at the phylum level after FMT. Gut microbiota composition at the phylum level **(A)** and abundances of individual bacteria **(B)** after FMT (on day 35 as indicated in **Figure 3A**). n = 4 in control group. n = 5 in ABX, ABX+BALB/c FMT, ABX+C57BL/6 groups. *P < 0.05. **P < 0.01.

To investigate the effect of FTM on the gut microbiota composition, gut microbiota profiles were assessed at the phylum, family, and genus levels at day 35. In mice of each treatment group, the gut microbiota comprised five phyla, including Bacteroidetes, Firmicutes, Proteobacteria, Saccharibacteria, and Verrucomicrobia (**Figure 5A**). The Firmicutes/Bacteroidetes ratio was higher, albeit not significantly, in the control and ABX+BALB/c FMT groups than in the ABX and ABX+C57BL/6 FMT groups (**Figure S1**). The abundances of Proteobacteria and Saccharibacteria were comparable among the control, ABX+BALB/c FMT, and ABX+C57BL/6 FMT groups but significantly higher than those in the ABX group. The abundance of Deferribacteres was significantly lower in the ABX and ABX+C57BL/6 FMT groups than in the control group. However, in the ABX+BALB/c FMT group, the abundance of Deferribacteres was even higher than that in the control group. The abundance of Tenericutes was significantly decreased in the ABX group but was restored in the ABX+BALB/c FMT and ABX+C57BL/6 FMT groups (**Figure 5B**).

**Figure 6:**
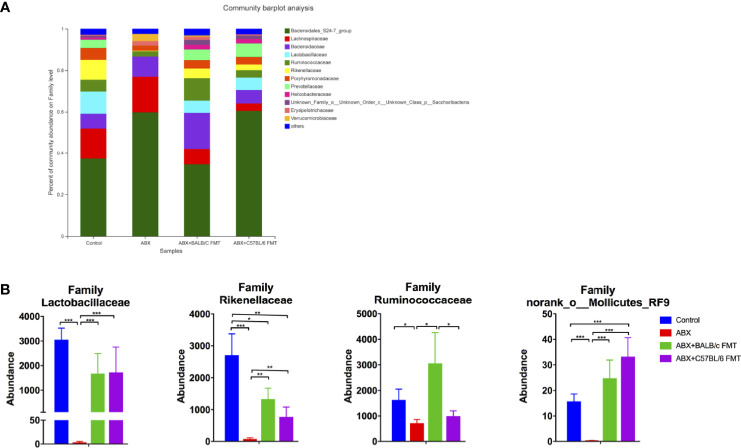
Comparison of the gut microbiota composition at the family level after FMT. Gut microbiota composition at the family level **(A)** and abundances of individual bacteria **(B)** after FMT (on day 35 as indicated in **Figure 3A**). n = 4 in control group. n = 5 in ABX, ABX+BALB/c FMT, ABX+C57BL/6 groups. *P < 0.05, **P < 0.01, ***P < 0.001.

At the family level, the gut microbiota profiles were shifted in the ABX, ABX+BALB/c FMT, and ABX+C57BL/6 FMT groups compared with the control group (**Figure 6A**). ABX treatment reduced the abundance of most families, including Lactobacillaceae, Rikenellaceae, Ruminococcaceae, and norank_o_Mollicutes_RF9. FMT restored the abundance of these families, but patterns differed between the ABX+BALB/c FMT and ABX+C57BL/6 FMT groups. In both the ABX+BALB/c FMT and ABX+C57BL/6 FMT groups, the abundance of Lactobacillaceae was restored to a level comparable to that in the control group. However, the abundance of Rikenellaceae remained significantly lower than that in the control group after FMT. The abundance of Ruminococcaceae was higher in the ABX+BALB/c FMT group than in the ABX+C57BL/6 FMT group. The abundance of norank_o_Mollicutes_RF9 in the ABX+C57BL/6 FMT group was even higher than that in the control group (**Figure 6B**).

**Figure 7:**
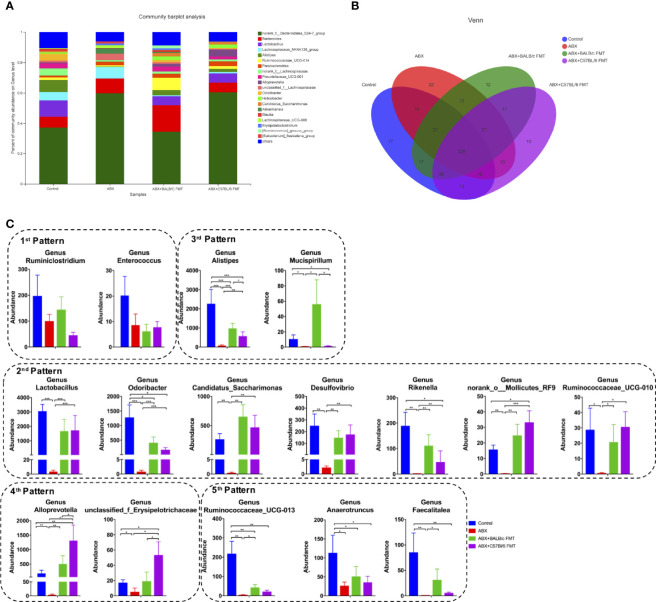
Comparison of the gut microbiota composition at the genus level after FMT. Gut microbiota composition at the genus level **(A)**, a 4-way Venn diagram **(B)** and abundances of individual bacteria **(C)** after FMT (on day 35 as indicated in **Figure 3A**). n = 4 in control group. n = 5 in ABX, ABX+BALB/c FMT, ABX+C57BL/6 groups. *P < 0.05, **P < 0.01, ***P < 0.001.

At the genus level, the gut microbiota profiles were shifted even more obviously after ABX treatment and FMT (**Figure 7A**). The shared and unique OTUs was shown in a 4-way Venn diagram (**Figure 7B**). We detected five distinct response patterns to ABX treatment and FMT at the genus level (**Figure 7C**). Genera in the 1^st^ pattern, including *Ruminiclostridium* and *Enterococcus*, showed no significant shift after ABX treatment and FMT, suggesting that the colonization of these commensal bacteria is very stable and not affected by ABX treatment and FMT. Genera exhibiting the 2^nd^ pattern were ablated by ABX treatment and restored by FMT, with no significant difference between the ABX+BALB/c FMT and ABX+C57BL/6 FMT groups. The 2^nd^ pattern included *Lactobacillus*, *Odoribacter*, *Candidatus Saccharimonas*, *Desulfovibrio*, *Rikenella*, *norank_Molicutes_RF9*, and *Ruminococcaceae_UCG-010.* These commensal bacteria were sensitive to ABX treatment and abundant in the fecal microbiota of both BALB/c mice and C57BL/6 mice. In the 3^rd^ pattern, genera were ablated by ABX treatment and restored by FMT, but their abundances were significantly higher in the ABX+BALB/c FMT group than in the ABX+C57BL/6 FMT group. The 3^rd^ pattern included *Alistipes*, *Ruminiclostridium*, *Mucispirillum*, *Alloprevotella*, and *unclassified_f_Erysipelotrichaceae*. In the 4^th^ pattern, genera were ablated by ABX treatment and restored by FMT, but their abundances were significantly lower in the ABX+BALB/c FMT group than in the ABX+C57BL/6 FMT group. The 4^th^ pattern included *Alloprevotella* and *unclassified_f_Erysipelotrichaceae*. Genera exhibiting the 5^th^ pattern were ablated by ABX treatment, but FMT had only a limited restorative effect, with no significant difference between the ABX+BALB/c FMT and ABX+C57BL/6 FMT groups. The 5^th^ pattern included *Ruminococcaceae_UCG-013, Anaerotruncus*, and *Faecalitalea.* These data showed that commensal bacteria from fecal microbiota do not completely, but selectively colonize the guts of recipient mice.

### Gut Microbiota Depletion and FMT Modulate HBV Infection Outcomes in BALB/c Mice

**Figure 8:**
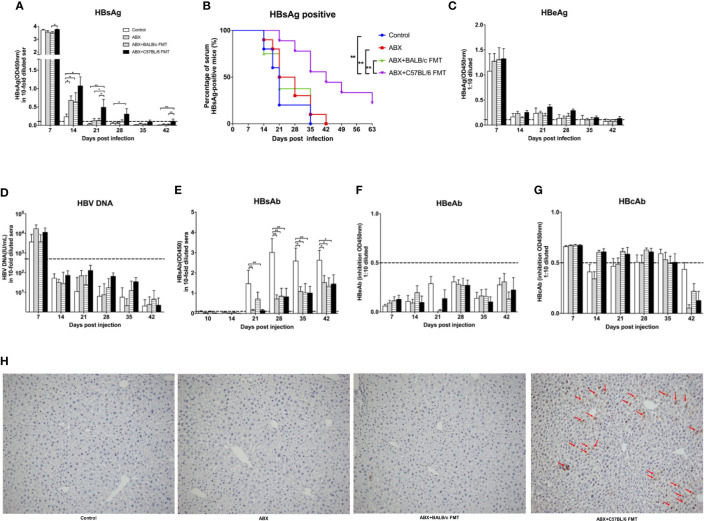
ABX treatment and FMT alter HBV infection outcomes in BALB/c mice. After gut microbiota reconstitution, BALB/c mice were hydrodynamically injected with pAAV/HBV1.2 (as indicated in **Figure 3A**). Serum levels of HBsAg **(A)**, HBeAg **(C)**, HBV DNA **(D)**, HBsAb **(E)**, HBeAb **(F)**, HBcAb **(G)** were measured at the indicated time points. **(B)** Kaplan–Meier curve showing the percentage of HBsAg-positive mice. Cutoff values are shown as dotted lines. **(H)** Mice were euthanized at 56 dpi, and HBcAg expression in the liver was measured by IHC staining. The HBcAg positive hepatocytes was labeled with red arrows. n = 10/group. *P < 0.05, **P < 0.01.

To evaluate the role of microbiota depletion and reconstitution during HBV infection, mice were hydrodynamically injected with the HBV plasmid pAAV/HBV1.2 on day 48 (**Figure 3A**). At 7 dpi, serum HBsAg levels were comparable among the control, ABX, and ABX+BALB/c FMT groups but were significantly higher in the ABX+C57BL/6 FMT group than in the ABX+BALB/c FMT group. At 14 dpi, serum HBsAg levels in the ABX, ABX+BALB/c FMT, and ABX+C57BL/6 FMT groups were significantly higher than those in the control group. However, after 21 dpi, only the HBsAg level remained higher in the ABX+C57BL/6 FMT group than in the control group. HBsAg was cleared within 21 days in the control group but persisted for 35, 42, and >63 days in the ABX+BALB/c FMT, ABX, and ABX+C57BL/6 FMT groups, respectively (**Figures 8A, B**).

Serum HBeAg levels were comparable among the four groups at 7 dpi and rapidly decreased after 14 dpi in all mice (**Figure 8C**). HBV DNA was detected in all mice at 7 dpi and disappeared after 14 dpi, with no significant differences among the four groups (**Figure 8D**). HBsAb was detected after 21 dpi, and the HBsAb level was significantly higher in the control group than in the three other groups (**Figure 8E**). HBeAb and HBcAb levels were comparable among the four groups (**Figures 8F, G**). At 56 dpi, HBcAg was undetectable in livers of mice from the control, ABX, and ABX+BALB/C FMT groups but was still detectable in livers of mice from the ABX+C57BL/6 FMT group (**Figure 8H**).

These results indicated that gut microbiota depletion can extend the duration of HBV infection in mice. Prolonged HBV infection due to gut microbiota depletion could not be reversed by BALB/c FMT and was even further prolonged by C57BL/6 FMT.

### Gut Microbiota Depletion and FMT Modulate the T-Cell Response in the Liver

**Figure 9:**
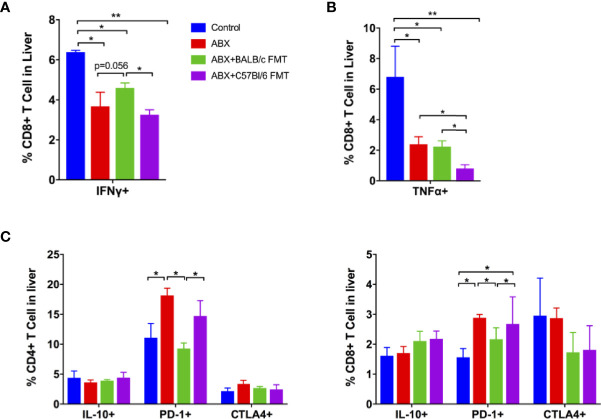
ABX treatment and FMT regulate the T-cell response to HBV infection. Lymphocytes were isolated from the liver at 42 dpi and stimulated with a CD8+ T-cell epitope. The expression of intracellular IFNγ **(A)** and TNFα **(B)** on CD8+ cells was measured by FACS. **(C)** The expression of intracellular IL-10, surface PD-1, and CTLA-4 on non-stimulated CD4+ and CD8+ T cells was measured by FACS. n = 10/group. *P < 0.05, **P < 0.01.

Specific T-cell responses in the liver were assessed at 42 dpi. After stimulation with CD8+ T cell epitope, the proportion of CD8+IFNγ+ T cells was decreased in the ABX, ABX+BALB/c FMT, and ABX+C57BL/6 FMT groups. FMT from BALB/c mice upregulated the proportion of CD8+IFNγ+ T cells after ABX; however, the effect was not significant. The proportion of CD8+IFNγ+ T cells was significantly higher in the ABX+BALB/c FMT group than in the ABX+C57BL/6 FMT group (**Figure 9A**). The proportion of CD8+TNFα+ T cells was decreased in the ABX, ABX+BALB/c FMT, and ABX+C57BL/6 FMT groups compared to the control group. BALB/c FMT had no effect on the production of TNFα in CD8+ T cells, but FMT from C57BL/6 mice significantly decreased TNFα production in CD8+ T cells compared to ABX and ABX+BALB/c FMT (**Figure 9B**). The expression of IL-17 and granzyme B in CD8+T cells was comparable among the four groups (**Figure S2**).

Finally, we measured the expression of inhibitory immune molecules on T cells in the liver. We observed no differences in the expression of IL-10 and CTLA-4 on CD4+ and CD8+ T cells among the four groups. PD-1 expression on CD4+ and CD8+ T cells was enhanced in the ABX and ABX+C57BL/6 FMT groups (**Figure 9C**). These data indicated that gut microbiota depletion impairs specific T-cell responses in the liver and that these responses can be restored partially by BALB/c FMT, but are further inhibited by C57BL/6 FMT.

## Discussion

This study showed that HBV infection is transient in BALB/c mice but can be persistent in C57BL/6 mice, which is consistent with findings in a previous study (Huang et al., 2006). The outcomes of HBV infection also differ in various substrains of C57BL/6 mice (Wang et al., 2018). The genetic differences have been identified between C57BL/6J and C57BL/6N strains (Simon et al., 2013), and the gut microbiota effects the differential phenotypes of colorectal cancer model in the two strains (Moskowitz et al., 2019). It is reasonable to hypothesize that HBV infection outcomes are related to the gut microbiota composition in different mouse strains. In this study, although the abundance and α-diversity of commensal bacteria were comparable in naïve BALB/c and C57BL/6 mice, their β-diversity and composition displayed marked differences. Firmicutes and Bacteroidetes are the dominant phyla in both human and mouse gut microbiota. The Firmicutes/Bacteroidetes ratio correlates with the progression of some chronic diseases. In our previous study, HBV infection elicited an increase in the Firmicutes/Bacteroidetes ratio in both transient and persistent HBV infection C57BL/6 mouse models, but the ratio was significantly higher in mice with transient infection than in those with persistent infection (Zhu et al., 2019). In this study, the Firmicutes/Bacteroidetes ratio was higher in naïve BALB/c mice than in naïve C57BL/6 mice, and HBV infection was transient in BALB/c mice but persistent in C57BL/6 mice. This is consistent with the finding that a higher Firmicutes/Bacteroidetes ratio is beneficial for HBV clearance. It is not well understood how the Firmicutes/Bacteroidetes ratio affects host immunity and metabolism. Increasing evidence indicates that the Firmicutes/Bacteroidetes ratio is related to the production of short-chain fatty acids (SCFAs) (Santos-Marcos et al., 2019), which modulate regulatory T cell (Treg) development and proliferation (Arpaia et al., 2013; Furusawa et al., 2013). Its association with the immune response during HBV infection requires further study.

The colonization of gut microbiota is influenced by multiple factors, including age, health status, environment, and diet. In this study, we distinguished five response patterns after FMT at the genus level. Although several genera were insensitive to ABX treatment, most commensal bacteria were depleted by the antibiotic conditioning regimen. In the 2^nd^ response pattern, FMT restored the abundance of part of the commensal bacteria, regardless of the fecal microbiota donor genotype. However, the existence of the 5^th^ pattern, in which genera were depleted by ABX treatment and FMT only had a limited restorative effect, indicated that commensal bacteria introduced *via* FMT cannot completely colonize the gut of the new host, regardless of the donor genotype. The mechanisms of selective colonization of commensal bacteria from fecal microbiota in a new host are not well understood. One possible explanation is that antibiotic treatment impairs the gut barrier function (Guo et al., 2021) and IgA production in the gut (Tian et al., 2009), which both play a critical role in the selection of commensal bacteria (Donaldson et al., 2018; Paone and Cani, 2020).

The gut microbiota modulates both innate and adaptive immunity of the host and protects against pathogen infection. In this study, ABX treatment enhanced PD-1 expression and inhibited specific T-cell responses, which in turn prolonged HBV infection, in BALB/c mice. FMT from BALB/c donor mice reversed PD-1 expression but did not restore the disrupted T-cell responses, despite the slightly increased IFN-γ production. As a result, HBV infection was not resolved as quickly in mice of the ABX+BALB/c FMT group as in naïve mice. One issue that should be addressed is that long-term antibiotic treatment may have side effects other than gut microbiota depletion. Increasing evidence shows that some antibiotics can directly suppress T-cell responses independently of microbiota dysbiosis. For example, linezolid, a ribosome-targeting antibiotic, impairs T-cell effector function by blocking mitochondrial protein synthesis (Almeida et al., 2021). In this study, six antibiotics were used to deplete the gut microbiota. Potential direct effect of these antibiotics on T-cell function should be further studied.

FMT can reconstitute the gut microbiota and alter host susceptibility to liver diseases. (Llopis et al., 2016). In this study, HBV infection was persistent in C57BL/6 mice, and fecal microbiota from C57BL/6 mice prolonged HBV infection in recipient BALB/c mice. Cytokine production by specific T cells in C57BL/6 fecal microbiota recipient mice was even lower than that in mice from the ABX group (**Figure 8A**). These results demonstrated that gut microbiota from C57BL/6 mice suppressed the HBV-specific immune response and promoted HBV infection persistence in mice. It is expected that commensal bacteria in the 4^th^ pattern contributed to the impairment of T cells response and HBV infection persistence, and genera in the 3^rd^ pattern corrected to robust T cells response and HBV clearance (**Figure 7C**). However, how individual bacterial species modulate the host immune response should be further studied. The abundance of *Alistipes* is decreased in patients with liver cirrhosis and correlates with disease progression from compensation to decompensation (Iebba et al., 2018; Parker et al., 2020). *Alistipes* can produce SCFAs, which modulate Treg and Th17 cell responses and suppress anti-inflammatory cytokine production (Parker et al., 2020). The abundance of *Mucispirillum* is associated with IgA production (Bunker et al., 2015) and contributes to the development of extrathymically generated Treg cells (Campbell et al., 2018).

The limitations of this study should be noted. First, we did not identify the individual bacterial species that affect HBV infection outcomes. Colonization of single species into germ-free mice should provide some insights in this regard. Second, the mechanism underlying the regulation of the host immune response in the liver by FMT was not unraveled. Potential regulatory factors, including SCFAs, Tregs, and IgA, should be further assessed. Hence, whether and how individual bacterial species regulate the immune response to HBV infection remains to be studied. Another issue that remains to be addressed is the effect of BALB/c FMT on HBV infection outcomes in C57BL/6 mice. Based on the study results, it is reasonable to hypothesize that BALB/c FMT would accelerate HBV clearance in C57BL/6 mice. However, we have in fact transferred fecal microbiota from naïve BALB/c mice into ABX-treated C57BL/6 mice and then established HBV infection. We found that BALB/c FMT did not enhance specific T-cell responses or affect HBV infection outcomes in the C57BL/6 mice (data not shown). This suggests that the effect of FMT at least in part depends on the host genetic background. This may explain the inconsistent results obtained in clinical trials applying FMT to treat patients with advanced liver diseases (Vaughn et al., 2019).

In conclusion, this study demonstrated that: 1) HBV infection outcomes in mice are affected by the host genetic background and gut microbiota composition, 2) the host genetic background shapes the gut microbiota composition, and commensal bacteria from FMT do not completely, but selectively colonize the gut of the new host, and 3) FMT can modulate the host immune response and alter host susceptibility to HBV infection. Thus, our data indicate the critical role of the gut microbiota in HBV infection outcomes. These findings are potentially helpful for the development of novel therapeutic strategies for chronic HBV infection.

## Data Availability Statement

The datasets presented in this study can be found in online repositories. The names of the repository/repositories and accession number(s) can be found below: https://www.ncbi.nlm.nih.gov/, SRP174629.

## Ethics Statement

The animal study was reviewed and approved by Institutional Animal Care and Use Committee at Tongji Medical College, Huazhong University of Science and Technology.

## Author Contributions

Study concept and design (JW and BW), acquisition of data (JW and XZho), analysis and interpretation of data (XL, WG, QZ, and BZ), drafting of the manuscript (JW and BW), critical revision of the manuscript for important intellectual content (XZhe), administrative, technical, or material support, study supervision (YL and DY).

## Funding

This work was supported by the National Science and Technology Major Project for Infectious Diseases of China (2017ZX10304402-002-005), the Chinese National Key Technology R&D Program (2015BAI09B06), and National Natural Science Foundation of China (grant numbers 81501748). The funders had no role in study design, data collection and analysis, decision to publish, or preparation of the manuscript.

## Conflict of Interest

The authors declare that the research was conducted in the absence of any commercial or financial relationships that could be construed as a potential conflict of interest.

## Publisher’s Note

All claims expressed in this article are solely those of the authors and do not necessarily represent those of their affiliated organizations, or those of the publisher, the editors and the reviewers. Any product that may be evaluated in this article, or claim that may be made by its manufacturer, is not guaranteed or endorsed by the publisher.

## Abbreviations

HBV, hepatitis B virus; ABX, broad-spectrum antibiotics; FMT, fecal microbiota transplantation; dpi, day post infection; OTUs, operational taxonomic units; SCFA, short-chain fat acid; Treg, regulatory T cells; HBsAg, hepatitis B virus surface antigen; HBeAg, hepatitis e antigen; HBcAg, hepatitis B virus core antigen; HBsAb, hepatitis B virus surface antibody; HBeAb, hepatitis B virus e antibody; HBcAb, hepatitis B virus core antibody.
