# Supplementary material for: Fecal Microbiota Transplantation Alters the Outcome of Hepatitis B Virus Infection in Mice
Source: Front Cell Infect Microbiol. 2022 May 4;12:844132. doi: 10.3389/fcimb.2022.844132 (PMC9114794; doi:10.3389/fcimb.2022.844132)
Supplement: Supplementary file 1 [file DataSheet_1.pdf]

Fig S1

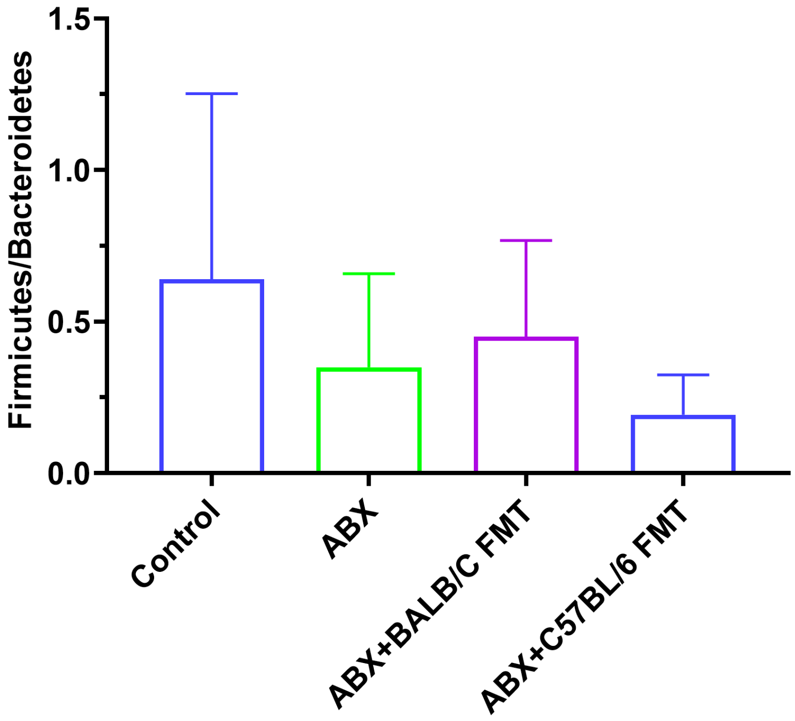

Fig.S1. The ratio of Firmicutes/Bacteroidetes after FMT (on day 35 as indicated in Fig. 3A).

Fig S2

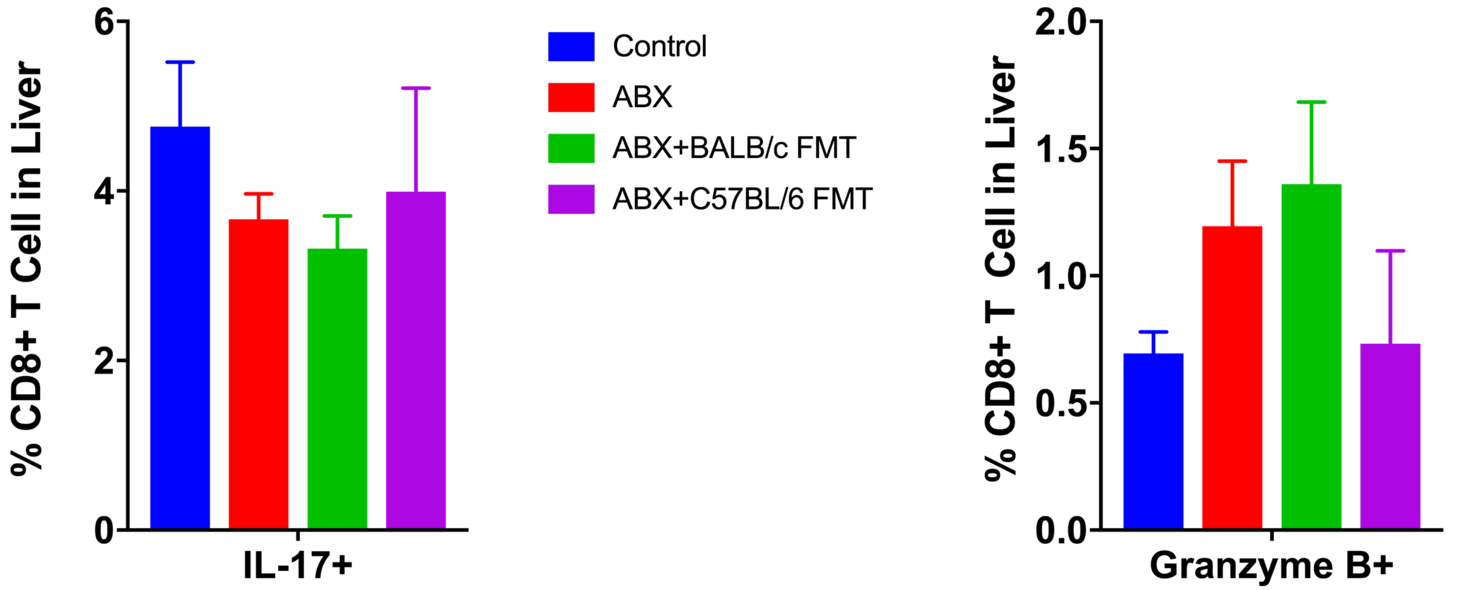

Fig. S2. ABX treatment and FMT regulate the T-cell response to HBV infection. Lymphocytes were isolated from the liver at 42 dpi and stimulated with a CD8+ T cell epitope. The expression of intracellular IL-17 and granzyme B on CD8+ cells was measured by FACS.
