# Supplementary material for: Fecal Microbiota Transplantation Alters the Outcome of Hepatitis B Virus Infection in Mice
Source: Front Cell Infect Microbiol. 2022 May 4;12:844132. doi: 10.3389/fcimb.2022.844132 (PMC9114794; doi:10.3389/fcimb.2022.844132)
Supplement: Supplementary file 2 [file DataSheet_2.pdf]

Supplemental material

Animal facility protocol

Genus: *Mus*

Species: *musculus*

Strain: *C57BL/6J and BALB/c*

Approximate age: 5-7 weeks when purchased from vendor, and bred at new facility at least 1 week prior to study.

Sex: male

Bacteriological status: *specific pathogen free*

Source: Hunan SAJ Laboratory Animal Co., Ltd. (Hunan, China)

Location where manipulation will be conducted: Experiment animal center, Tongji Medical College, Huazhong University of Science and Technology.

Anesthesia and euthanasia: Mice were anesthetized with 2%-4% diethyl ether, and euthanized by cervical dislocation.

Experimental design and animal procedures were shown as follow:

Part 1. *C57BL/6J and BALB/c*, 6-8 weeks, were hydrodynamic injected with pAAV/HBV1.2. Bleed the mice from retro-orbital sinus once a week, euthanize the mice at 9 weeks after infection.

Part 2. Fresh fecal pellets were collected from naïve *C57BL/6J and BALB/c* (6-8 weeks). 16s RNA sequencing was performed.

Part 3. The gut microbiota depletion and FMT procedures were showed in the following flowchart.

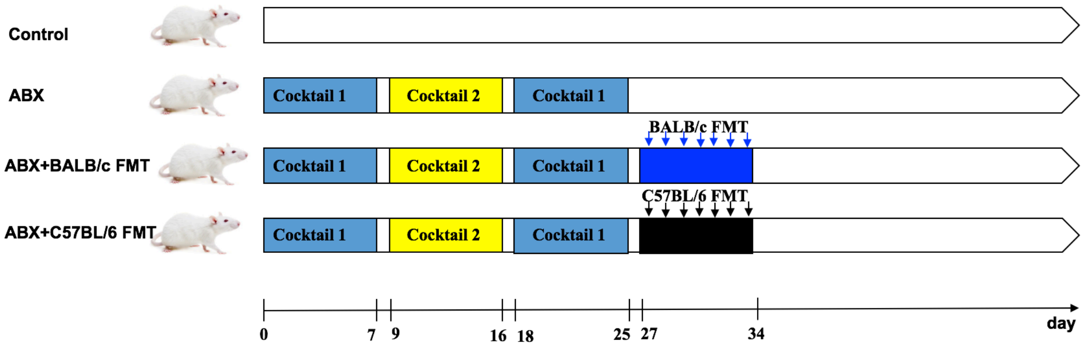

Fecal pellets were collected at day 25 and day 35, and 16s RNA sequencing was performed.

Part 4. The experimental design was showed in the following flowchart (Fig 3A).

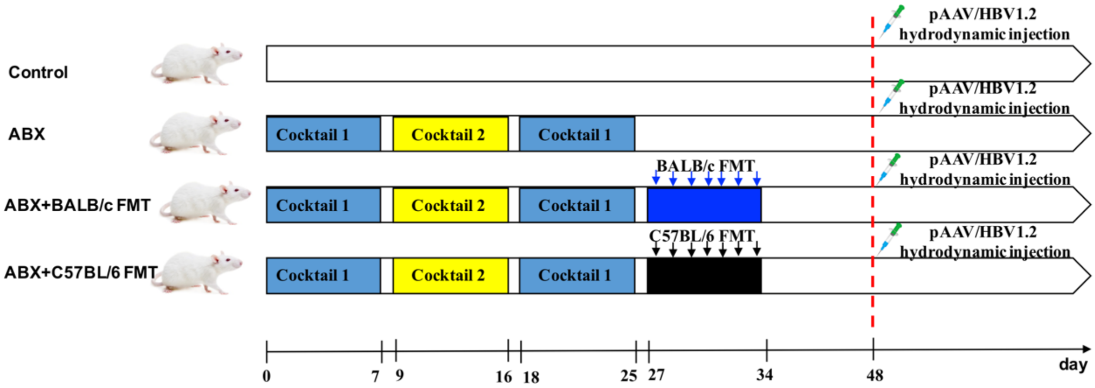

Bleed the mice from retro-orbital sinus once a week after infection, euthanize the mice at 6 weeks after infection.

Bioinformatic analysis

Raw FASTQ files were de-multiplexed using an in-house perl script, and then quality-filtered by fastp version 0.19.6 and merged by FLASH version 1.2.7 with the following criteria: (i) the 300 bp reads were truncated at any site receiving an average quality score of <20 over a 50 bp sliding window, and the truncated reads shorter than 50 bp were discarded, reads containing ambiguous characters were also discarded; (ii) only overlapping sequences longer than 10 bp were assembled according to their overlapped sequence. The maximum mismatch ratio of overlap region is 0.2. Reads that could not be assembled were discarded; (iii) Samples were distinguished according to the barcode and primers, and the sequence direction was adjusted, exact barcode matching, 2 nucleotide mismatch in primer matching. Then the optimized

sequences were clustered into operational taxonomic units (OTUs) using UPARSE 7.1 with 97% sequence similarity level. The most abundant sequence for each OTU was selected as a representative sequence. The taxonomy of each OTU representative sequence was analyzed by RDP Classifier version 2.2 against the 16S rRNA gene database (Silva v138) using confidence threshold of 0.7.

As previous report(Chen et al., 2020), sequence reads were quality checked by Trimmomatic. OTUs were generated by QIIME and taxonomies were classified using the Ribosomal Database Project (RDP) classifier script (version 2.2). The α-diversity, including OTU counts, Chao1 community richness, and Shannon–Weaver index values was calculated by mothur (version v.1.30.1). The β-diversity, as determined by Bray-Curtis dissimilarity, was analyzed by vegan package in R.

Reference:

Chen, Y., Qiu, X., Wang, W., Li, D., Wu, A., Hong, Z., et al. (2020). Human papillomavirus infection and cervical intraepithelial neoplasia progression are associated with increased vaginal microbiome diversity in a Chinese cohort. *BMC Infect Dis* 20**,** 629. doi:10.1186/s12879-020-05324-9
